# One-Year Outcomes of Long Coronary Drug-Eluting Stents (≥40 MM) in Patients With Diffuse Coronary Artery Disease: Findings From a Tertiary Care Hospital in North India

**DOI:** 10.7759/cureus.59611

**Published:** 2024-05-03

**Authors:** Suraj Khanal, Ayush Agarwal, Basant Kumar

**Affiliations:** 1 Cardiology, Post Graduate Institute of Medical Education and Research (PGIMER), Chandigarh, IND; 2 Internal Medicine, Post Graduate Institute of Medical Education and Research (PGIMER), Chandigarh, IND

**Keywords:** target lesion failure rate, long des, drug-eluting stents, percutaneous coronary intervention, coronary artery disease

## Abstract

Background and objective

Diffuse coronary artery disease (CAD) is associated with extensive involvement of coronary arteries, necessitating the use of long (≥40 mm) drug-eluting stents (DES) based on the lesion length. However, these long DES can lead to complications such as in-stent restenosis (ISR) and stent thrombosis. This study aimed to assess the safety, efficacy, and one-year clinical outcomes of using long DES in patients with diffuse CAD undergoing PCI at a tertiary care hospital in north India.

Methodology

Patients with diffuse CAD undergoing PCI with long DES between January 2017 and June 2022 were included in the study. Baseline characteristics were recorded, and patients were followed up telephonically or in the outpatient department (OPD) at one, three, six, and 12 months following the PCI. The primary endpoint was the target lesion failure (TLF) rate, with secondary endpoints constituting all-cause mortality, major adverse cardiovascular events (MACE), subacute stent thrombosis, and ISR.

Results

A total of 200 patients were recruited and followed up for one year. The median age of the patients was 58 years (range: 48.25-63 years), and 82% were men. The most frequently stented artery was the left anterior descending (LAD, 48%), followed by the right coronary artery (RCA, 36%). A total of 388 stents (mean: 1.94 ±0.79) were implanted, including both long and short stents. The mean length and diameter of long stents were 43.64 ±3.58 mm and 3 ±0.37 mm, respectively. At the one-year follow-up, patients undergoing PCI with long DES ≥40 mm had an overall TLF rate of 5%, all-cause mortality of 6% (12 patients), MACE of 6% (12 patients), subacute stent thrombosis of 4% (eight patients), and ISR of 1% (two patients). A large proportion of patients (90%) had an uneventful follow-up of up to a year. At the one-year follow-up, all 10 (5%) patients with a primary outcome had a smaller stent diameter than those without a primary outcome (2.5 ±0.25 mm vs. 3.03 ±0.35 mm, p=0.015).

Conclusions

Our results suggest that using extremely long stents (>40 mm) for diffuse coronary lesions is safe, efficacious, and associated with relatively low event rates. In addition, the stent diameter has a substantial correlation with the primary outcome. Further studies with larger sample sizes as well as longer follow-up periods are required to validate our findings.

## Introduction

Coronary artery disease (CAD) is a major health concern globally, and its incidence has been steadily rising over the years. In 2015, CAD was responsible for 8.9 million deaths and 164.0 million disability-adjusted life years (DALYs) worldwide [1]. In India, CAD is a serious and growing public health concern, particularly in individuals over the age of 20 years [2]. The formation of atherosclerotic plaque, which narrows the vessel lumen and obstructs blood flow, is a defining feature of CAD pathogenesis. As the plaque grows, the artery lumen gradually narrows, causing ischemia. Diabetes, hypertension, hypercholesterolemia, smoking, obesity, and physical inactivity are all linked to an increased risk of CAD. Nowadays, percutaneous coronary intervention (PCI) using drug-eluting stents (DES) is a widely used and successful therapeutic choice for managing coronary lesions [3]. Coronary lesions are classified into three types based on their size: focal or type A lesions (<10 mm long), unfavorable or type B lesions (10-20 mm long), and diffuse or type C lesions (>20 mm long) [4]. Classifying a lesion as "long" is subjective, and no consensus exists regarding the same. However, according to the Synergy Between PCI With Taxus and Cardiac Surgery (SYNTAX) score, a more relevant lesion categorization approach, lesion lengths of more than 20 mm are a greater risk factor [5].

Diffuse long coronary artery lesions present a therapeutic challenge since they frequently necessitate the placement of several overlapping stents and are associated with poor clinical outcomes. A study found that 20% of all PCI patients have diffuse long coronary artery lesions [6-7]. Multiple overlapping stents used to cover long lesions were found to increase the risk of stent fracture, restenosis, and stent thrombosis, notably in the early-generation DES era [8-9]. Furthermore, a study of over 8000 patients found that stent lengths greater than 32 mm were associated with increased rates of target vessel revascularization (TVR) and stent thrombosis in individuals treated with first-generation DES but not with second-generation DES [10]. Second-generation DES was developed to increase endothelial coverage while decreasing inflammation.

The new generation of DES has addressed the limitations of earlier generations by employing ultrathin struts and a more flexible stent design [11], thereby improving the overall prognosis of ST-segment elevation myocardial infarction (STEMI) patients [3]. In addition, PCI with long stents has a lower contrast volume, treatment duration, and fluoroscopy time compared to procedures using multiple shorter stents, thereby paving the way for using long DES in diffuse lesions [12]. As demonstrated in more recent studies, treating diffuse coronary lesions with very long DES (48 and 40 mm) is safe, effective, and associated with fairly low event rates, and involves fewer stents overall [13-14]. However, diffuse CAD is considered unfavorable for PCI, and data are scarce on the outcomes of PCI with very long DES (≥40 mm). Hence, this study aimed to assess the safety, efficacy, and one-year clinical outcomes of using long DES (≥40 mm) in patients with diffuse CAD undergoing PCI at a tertiary care hospital in north India.

## Materials and methods

Study design and setting

This was a prospective study conducted between January 2017 and June 2022 at the Post Graduate Institute of Medical Education and Research (PGIMER), Chandigarh, a tertiary care institution in Northern India. It involved patients of both sexes aged ≥18 years with diffuse CAD, who underwent PCI with a long (≥40 mm) DES and provided informed written consent. Patients with chronic kidney disease (CKD) or myxoid heart disease (MHD) were excluded from the study. Patients were also excluded if they had acute left ventricular failure (LVF), cardiogenic shock, or a high thrombus burden. These patients were excluded since they were at a higher risk, and the study intervention would be less effective for them. Patients who had an on-table death or who did not survive the procedure were also excluded from the study. The decision to implant a long DES (≥40 mm) was made by the primary operator. The study was conducted following the ethical principles outlined in the latest version of the Declaration of Helsinki, as well as the applicable good clinical practice guidelines, and approval from the institutional ethics committee was obtained via letter number INT/IEC/2023/00616 dated June 7, 2023.

Data collection

Baseline information such as gender, age, risk factors for CAD, clinical presentation, and angiographic severity of CAD were recorded. Procedural parameters such as total stents utilized, stent length and diameter, and type of DES were also obtained from medical records. Patients were followed up telephonically or in the outpatient department (OPD) at one, three, six, and 12 months following the PCI. During follow-up, patients had specific examinations such as fasting blood sugar (FBS), lipid profile, an electrocardiogram (ECG), and echocardiography as part of the standard institutional practices. Symptomatic patients underwent repeat coronary angiography (CAG).

Primary Endpoint

The primary endpoint was the target lesion failure (TLF) rate, defined as a composite of cardiac death, any myocardial infarction (not attributable to a non-target vessel), or any ischemia-driven revascularization of the target lesion (ID-TLR) within 12 months.

Secondary Endpoints

The secondary endpoints were all-cause mortality, major adverse cardiovascular events (MACE), subacute stent thrombosis, in-stent restenosis (ISR), etc. All-cause mortality was defined as death from any cause. MACE was defined as the composite of total death, myocardial infarction, coronary revascularization, stroke, and heart failure. Subacute stent thrombosis was defined as thrombosis occurring within 30 days following stent implantation. ISR was defined as the narrowing of the stent lumen resulting in anginal symptoms. Target lesion revascularization (TLR) was defined as any procedure performed to restore luminal patency following late luminal loss due to ISR. During the follow-up period, primary and secondary outcomes were assessed via physical examination, medical history, laboratory evaluation, ECG, and echocardiography.

Statistical analysis

Quantitative variables were presented as mean with standard deviation (SD). Frequencies and percentages were used to summarize categorical data. Univariate analysis was performed to determine the effects of factors affecting outcomes and presented as relative risk with a 95% confidence interval (CI). Multivariate analysis was done using generalized linear models. The variables with p-values <0.10 were included in multivariate analysis. Adjusted relative risk ratio with 95% CI was calculated. A p-value <0.05 was considered statistically significant. Statistical analysis was performed using SPSS Statistics (IBM Corp., Armonk, NY).

## Results

Baseline characteristics

The baseline characteristics of the study population are presented in Table 1.

**Table 1:** Baseline, angiographic, and procedural characteristics of the study population IQR: interquartile range; CAD: coronary artery disease; PCI: percutaneous coronary intervention; STEMI: ST-segment elevation myocardial infarction; NSTEMI: non-STEMI; LV: left ventricular; LAD: left anterior descending artery; LCx: left circumflex; RCA: right coronary artery

Variables	Values (N=200)
Gender, n (%)	
Male	164 (82)
Female	36 (18)
Median age (IQR), years	58 (48.25–63)
Clinical presentation, n (%)	
STEMI	36 (18)
NSTEMI	36 (18)
Diabetes mellitus	72 (36)
Hypertension	88 (44)
Chronic coronary syndrome	68 (34)
Unstable angina	60 (30)
LV dysfunction (EF <40%)	84 (42)
Previous PCI, n (%)	28 (14)
Angiographic severity of CAD (target vessel location), n (%)	
Single vessel disease	48 (24)
LAD	24 (50)
LCx	0 (0)
RCA	24 (50)
Double vessel disease	76 (38)
Triple vessel disease	76 (38)

The cohort comprised 164 men (82%) and 36 women (18%), with a median age of 58 years (range: 48.25-63 years). Diabetes was present in 72 (36%) patients, whereas hypertension, unstable angina, and chronic coronary syndrome were present in 88 (44%), 60 (30%), and 68 (34%) patients, respectively. The clinical presentation of PCI was STEMI or non-STEMI (NSTEMI) in 36 (18%) patients each; 84 (42%) patients had left ventricular (LV) dysfunction (ejection fraction <40%). Previous PCI was reported in 28 (14%) patients. Coronary angiogram (CAG) indicated single-vessel disease in 48 (24%) patients, whereas 76 (38%) patients had double-vessel disease while 76 (38%) had triple-vessel diseases. Baseline characteristics suggested male dominance, an older age group, and a complex disease presentation.

Procedural characteristics

The procedural characteristics of the study population are presented in Table 2.

**Table 2:** Procedural characteristics SD: standard deviation; LAD: left anterior descending artery; LCx: left circumflex; RCA: right coronary artery; IQR: interquartile range; DES: drug-eluting stent

Variables	Values
Total stents used (mean ±SD)	388 (1.94 ±0.79)
Number of long stents used	228
One long stent, n (%)	172 (86)
Two long stents, n (%)	28 (14)
Target vessel location for long stents, n (%)	
LAD	96 (48)
LCx	8 (4)
RCA	72 (36)
LAD and RCA	16 (8)
LAD and LCx	8 (4)
Stent length	
Median (IQR)	44 mm (40–48 mm)
Mean ±SD	43.64 ±3.58 mm
Maximum	50 mm
Stent diameter	
Median (IQR)	3 mm (2.75–3.38 mm)
Mean ±SD	3 ±0.37 mm
Maximum	4 mm
DES, n (%)	
Sirolimus-eluting long stent	172 (75.4)
Everolimus-eluting long stent	56 (24.6)

A total of 388 (mean 1.94±0.79) stents, comprising both long and short stents, were implanted. Overall, 228 long stents were used, with one long stent in 172 (86%) patients and two long stents in 28 (14%). Moreover, 160 (80%) patients had an additional shorter stent placed beside the long stent. Of note, 64 patients (32%) had a short stent and a long stent placed in the same artery, 52 of which overlapped with the long stent. Only four patients (2%) underwent overlapping with two long stents. Long stents were implanted in the LAD in 96 patients (48%), the RCA in 72 patients (36%), the left circumflex (LCx) in eight patients (4%), the LAD and RCA in 16 patients (8%), and the LAD and LCx in eight patients (4%). The stent length ranged from 40 to 48 mm, with a median of 44 mm and a mean of 43.64 ±3.58 mm. The longest stent length was 50 mm, utilized in four patients (2%). The median stent diameter was 3 mm (2.75-3.38 mm), while the mean was 3 ±0.37 mm. The maximum stent diameter was 4 mm; 172 (75.4%) of the long stents implanted were sirolimus-eluting stents, with 56 (24.6%) being everolimus-eluting stents. As part of the standard institutional practice, patients were prescribed aspirin SR 150 mg, clopidogrel 75 mg, and rosuvastatin 20 mg once daily till the end of the one-year follow-up.

Clinical outcomes

The one-year outcomes in the patients are presented in Table 3.

**Table 3:** Clinical outcomes (N=200) ISR: in-stent restenosis; MACE: major adverse cardiovascular events; CAG: coronary angiogram

Variables	Values, n (%)
All-cause mortality	10 (5)
Cardiac death (within one month after the procedure)	8 (4)
Probable subacute stent thrombosis	8 (4)
ISR	2 (1)
Any MACE	12 (6)
CAG on follow-up	4 (2)

One-year follow-up data was available for 184 patients (92%) evaluated in person in the OPD. Of the remaining 16 patients, eight (4%) developed probable subacute stent thrombosis leading to sudden cardiac death at home (within one month after the procedure), two (1%) had non-cardiovascular causes of death in the hospital (one from acute renal failure, and the other from gastroenteritis), and six (3%) were lost to follow-up. Four (2%) of 184 patients underwent repeat CAG due to symptoms. Of these, two patients underwent CAG at the six-month follow-up (one had 40% ISR in LAD stent and the other had a patent stent), and two patients underwent CAG at the one-year follow-up (both had focal ISR of 90%, which was treated with sirolimus-eluting balloon). The remaining 180 patients (90%) had an uneventful follow-up for up to one year. Overall, MACE was seen in 12 (6%) patients. At the one-year follow-up, patients undergoing PCI with long DES ≥40 mm had an overall TLF rate of 5%, all-cause mortality of 6%, MACE of 6%, subacute stent thrombosis of 4%, and ISR of 1%.

Multivariate analysis was employed to assess factors influencing the primary outcome of TLF at the one-year follow-up (Table 4).

**Table 4:** Factors affecting the primary outcome of TLF at one-year follow-up ^¥^The Mann-Whitney U test. ^¥¥^Fisher’s exact test. ^*^Statistically significant TLF: target lesion failure; SD: standard deviation; LV: left ventricle; DES: drug-eluting stent

Variables	P-value
Age (mean ±SD)^¥^	0.838
Sex (M/F)^¥¥^	1.000
Diabetes mellitus^¥¥^	1.000
Hypertension^¥¥^	1.000
LV dysfunction^¥¥^	<0.005*
Clinical presentation^¥¥^	1.000
Number of vessels stented^¥¥^	0.447
Type of DES^¥¥^	0.604
Number of long stents^¥¥^	1.000
Site of long stent implantation^¥¥^	0.150
Total stent length (mean ±SD)^¥^	0.268
Diameter of stents (mean ±SD)^¥^	0.015*

There was no statistically significant difference in age, sex, presence or absence of diabetes or hypertension, clinical presentation, number and site of vessel stented, type of DES, and length of stents between patients who had a primary outcome of TLF and those who were doing well up to one year. The only statistically significant difference (p<0.005) observed pertained to LV dysfunction and the diameter of long stents. At the one-year follow-up, all 10 (5%) patients with a primary outcome had a smaller stent diameter than those without a primary outcome and were doing well (2.5 ±0.25 mm vs. 3.03 ±0.35 mm, p=0.015).

## Discussion

CAD has a significant detrimental effect on people's health and quality of life [15]. According to studies, long coronary lesions account for 20% of CAD cases. They are classified as a high-risk lesion subtype due to the greater incidence of dissection, abrupt vascular closure, and restenosis compared to focal lesions [16-17]. However, clinical studies on long lesions differ greatly in their definition; some characterize it as >30 mm, while others define it as >40 mm long [17]. It is also widely acknowledged that PCI procedures have evolved rapidly along with continuing advances in medical technology [15]. However, interventional cardiologists find it difficult to decide whether to implant multiple short stents or a single long stent in challenging cases of CAD that encompass a wide variety of complicated lesions [18]. In contrast, diffuse CAD necessitates a longer stent to be deployed and has a poor prognosis.

DES has been used in clinical practice for almost two decades and involves a range of materials, polymers, and drug types. However, even with the advent of design and material enhancements that improve biocompatibility and antithrombotic qualities, additional clinical monitoring and follow-up are still necessary to assess their long-term safety and efficacy [3]. Even though long stents have been available for quite some time, there is limited information in the literature on the use of ultralong DES (≥40 mm) for long coronary lesions. In our study, patients undergoing PCI with long DES ≥40 mm had an overall TLF rate of 5% (10 patients), all-cause mortality of 6% (12 patients), MACE of 6% (12 patients), subacute stent thrombosis of 4% (eight patients), and ISR of 1% (two patients) at the one-year follow-up. Our single-center prospective real-world study conducted in northern India sheds light on the safety, efficacy, and one-year clinical outcomes of using long DES (≥40 mm) in patients with diffuse CAD.

The demographic characteristics of our study population, such as the mean age and gender distribution, were similar to those reported in previous studies [13,19]. Furthermore, our study cohort had a high prevalence of hypertension (44%), followed by diabetes (36%), chronic coronary syndrome (34%), and unstable angina (30%). These findings are consistent with previous research, which found a high prevalence of hypertension (54%), diabetes (46%), and chronic stable angina (29%) in the studied group [13-14]. In addition, our study included a higher proportion of sirolimus-eluting stents (75.4%) than everolimus-eluting stents (24.6%), which aligns with a prior study, where a higher percentage of sirolimus-eluting stents (62%) were utilized than everolimus-eluting stents (38%) [13].

The TLF rate in our study was 5% (10 patients), which represented cardiac deaths and TLR within 12 months. A previous study, which involved ultralong (≥40 mm) and ultrathin (60 μm) biodegradable polymer-coated sirolimus-eluting stents, reported a TLF rate of 6.1% (42 patients) at the one-year follow-up due to cardiac death, target vessel myocardial infarction, and TLR in nine (1.3%), 20 (2.9%), and 13 (1.9%) patients, respectively [11]. Another study that assessed the clinical outcomes of a single long (48 mm) DES with two overlapping DES found a TLF rate of 2.6% at one year in the single-long DES group vs. 4.1% in the overlapping DES group. Furthermore, after two years, TLF was 5.3% in the single-long DES group against 6.3% in the overlapping DES group [14]. In another study that investigated one-year clinical outcomes after PCI with very long (≥40 mm) DES, the TLF rate was 2.9% [13].

A previous study evaluating the one-year outcome of a long stent (32 or 38 mm) revealed a TLF rate of 3.1% [20]. The TLF rates of 2.6% to 6.1% reported in these studies align with our findings. Another study, using ultralong (≥40 mm) and ultrathin (60 μm) biodegradable polymer-coated sirolimus-eluting stents, revealed a TLR of 1.9% [11], which is consistent with our findings. The Percutaneous Treatment of LONG Native Coronary Lesions With Drug-Eluting Stent-IV (LONG-DES-IV) trial focused on long lesions (defined as >25 mm) [21]. The study used a 38-mm resolute zotarolimus-eluting stent, and the one-year TLF rate was 14%, substantially higher than the 5% reported in our study. Our study found low rates in terms of one-year outcomes, indicating the safety of using very long (≥40 mm) DES over one year.

The overall rate of MACE in this study was 6%, which is in line with previous studies that employed long DES (48 mm or >40 mm) and reported 6% MACE at a one-year follow-up [13-14]. A study comparing the primary outcome of multiple stents vs. single long stents in long coronary lesions found that the MACE rate increased significantly with multiple overlapping stenting compared to single long stenting (7.1% vs. 1.5%, p<0.001) [22]. Another study compared the one-year outcomes of patients with acute myocardial infarction who received a single long stent (stent length: ≥38 mm) versus those who received overlapping double short stents (individual stent lengths: <38 mm) and found that one-year MACE was comparable between the two groups (HR: 1.33; 95% CI: 0.80-2.24) [18]. Furthermore, according to the findings of a meta-analysis, DES has been linked to a reduced MACE, primarily through a reduced incidence of TLR without increased risk of stent thrombosis in high bleeding-risk PCI patients [23]. This study's MACE rate of 6% is consistent with previous reports demonstrating the safety of using long DES (≥40 mm) in patients with diffuse CAD.

We found a subacute stent thrombosis rate of 4% (eight patients), which was greater than the 2.2% (seven patients) reported in an earlier study [13]. Furthermore, in the same study, 17 (5.4%) patients had CAG during follow-up, with four (1.27%) having mild ISR and three (1%) requiring target vessel revascularization. In contrast, only four (2%) patients in our study underwent CAG during follow-up, and two (1%) had ISR. The ISR rate in our study was comparable with previous findings. Previous studies suggest that bifurcation stenting, longer stents, decreased left ventricular systolic function, and small stent dimensions (mean diameter: <3.0 mm) might all lead to increased stent thrombosis [11,24]. In this study, we used both sirolimus-eluting and everolimus-eluting stents and found no statistically significant association between the primary outcomes and type of DES. A previous study comparing event rates between sirolimus-eluting stents and everolimus-eluting stents supports these findings and demonstrated no statistically significant difference between the two types of DES in outcomes such as cardiac death (1.7% vs. 1.5%; p=0.911), stent thrombosis (2.5% vs. 1.7%; p=0.612), TLR (1% vs. 0.8%; p=0.878), and any MACE (7% vs. 4.1%; p=0.284) [13].

In our study, all 10 (5%) patients with a primary outcome up to one-year follow-up had a smaller stent diameter (2.5 ±0.25 mm), while the remaining patients who had no primary outcome and were doing well had a higher stent diameter (3.03 ±0.35 mm). This finding aligns with a previous study that found significant positive associations between stent diameter and TVR [continuous: odds ratio (OR): 0.485, 95% CI: 0.305-0.773, p=0.002). The piecewise linear regression model found that when the stent diameter ranged between 2.5 and 2.9 mm, the necessity for TVR decreased (OR: 0.01, 95% CI: 0.01-0.13, p<0.001) [19]. Furthermore, a previous study found that a larger stent with a diameter of less than 2.5 mm provided little protection [19]. According to research, the incidence of coronary perforation rose with the introduction of DES stents, particularly in chronic total occlusion, severe calcification, or eccentric lesions [25-26]. The association between the primary outcome and a smaller stent diameter may be attributed to a greater risk of restenosis in smaller reference vessels compared to larger reference vessels in diffuse CAD with long lesions. Furthermore, those with small-vessel disease are more likely to suffer from diabetes and have lesions that are longer and more convoluted. Our findings can help clinicians in their decision-making and perhaps improve outcomes for patients having PCI with long stents for diffuse coronary lesions.

Limitations of the study

This study has a few limitations, primarily its small sample size; further studies with sufficient power to validate these findings are required. Also, due to the short follow-up duration, the long-term clinical outcomes, MACE, and all-cause mortality in patients with very diffuse CAD following PCI with long DES could not be evaluated. We recommend conducting further studies with larger sample sizes and longer follow-up periods to confirm the findings of our study.

## Conclusions

Based on our findings, using extremely long stents (>40 mm) for diffuse coronary lesions is safe, effective, and associated with relatively low event rates. Furthermore, the stent diameter is significantly correlated with the primary outcome and may have a substantial impact on event rates following PCI in diffuse CAD with long lesions.

## References

[1] Zhang G, Yu C, Zhou M, Wang L, Zhang Y, Luo L (2018). Burden of Ischaemic heart disease and attributable risk factors in China from 1990 to 2015: findings from the Global Burden of Disease 2015 Study. BMC Cardiovasc Disord.

[2] Mavkar SS, Shukla MP (2024). Effect of Buteyko breathing technique as an adjunct to routine physiotherapy on pulmonary functions in patients undergoing off-pump coronary artery bypass surgery: a randomized controlled trial. Indian J Crit Care Med.

[3] Zeng Y, Xu J, Deng Y, Li X, Chen W, Tang Y (2024). Drug-eluting stents for coronary artery disease in the perspective of bibliometric analysis. Front Cardiovasc Med.

[4] Ryan TJ, Faxon DP, Gunnar RM (1988). Guidelines for percutaneous transluminal coronary angioplasty. A report of the American College of Cardiology/American Heart Association Task Force on Assessment of Diagnostic and Therapeutic Cardiovascular Procedures (Subcommittee on Percutaneous Transluminal Coronary Angioplasty). Circulation.

[5] Sianos G, Morel MA, Kappetein AP (2005). The SYNTAX Score: an angiographic tool grading the complexity of coronary artery disease. EuroIntervention.

[6] Gwon HC, Jeon DW, Kang HJ (2017). The practice pattern of percutaneous coronary intervention in Korea: based on year 2014 cohort of the Korean Percutaneous Coronary Intervention (K-PCI) Registry. Korean Circ J.

[7] Bourassa MG, Lespérance J, Eastwood C, Schwartz L, Côté G, Kazim F, Hudon G (1991). Clinical, physiologic, anatomic and procedural factors predictive of restenosis after percutaneous transluminal coronary angioplasty. J Am Coll Cardiol.

[8] Iakovou I, Schmidt T, Bonizzoni E (2005). Incidence, predictors, and outcome of thrombosis after successful implantation of drug-eluting stents. JAMA.

[9] Doi H, Maehara A, Mintz GS (2009). Classification and potential mechanisms of intravascular ultrasound patterns of stent fracture. Am J Cardiol.

[10] Choi IJ, Koh YS, Lim S (2014). Impact of the stent length on long-term clinical outcomes following newer-generation drug-eluting stent implantation. Am J Cardiol.

[11] Sinha SK, Aggarwal P, Pandey U, Razi M, Kumar A, Krishna V (2021). Ultrathin (60 μm), ultralong (≥40 mm) sirolimus-eluting stent: study of clinical and safety profiles among real-world patients. Anatol J Cardiol.

[12] Jurado-Román A, Abellán-Huerta J, Requena JA (2019). Comparison of clinical outcomes between very long stents and overlapping stents for the treatment of diffuse coronary disease in real clinical practice. Cardiovasc Revasc Med.

[13] Rajesh GN, Sulaiman S, Vellani H, Sajeev CG (2018). One-year clinical outcome of percutaneous coronary intervention with very long (≥ 40mm) drug-eluting stent. Indian Heart J.

[14] Sim HW, Thong EH, Loh PH (2020). Treating very long coronary artery lesions in the contemporary drug-eluting-stent era: single long 48 mm stent versus two overlapping stents showed comparable clinical outcomes. Cardiovasc Revasc Med.

[15] Bai X, Shen C, Zhang W, Yu T, Jiang J (2023). Efficacy and risks of drug-coated balloon treatment for coronary artery disease: a meta-analysis. Heliyon.

[16] Oh PC, Han SH (2019). Diffuse long coronary artery disease is still an obstacle for percutaneous coronary intervention in the second-generation drug-eluting stent era?. Korean Circ J.

[17] Ragosta M (2020). Stenting long coronary lesions: can one stent do the job of two?. Cardiovasc Revasc Med.

[18] Lee DH, Oh S, Kim MC (2023). Comparative treatment outcomes of a single long stent vs. overlapped short stents in acute myocardial infarction. Front Cardiovasc Med.

[19] Xu T, Feng B, Zheng Z (2021). Association of stent diameter and target vessel revascularization in patients undergoing percutaneous coronary intervention: a secondary retrospective analysis based on a Chinese cohort study. BMC Cardiovasc Disord.

[20] Teirstein PS, Stone GW, Meredith IT (2011). Platinum chromium everolimus-eluting stent in long coronary lesions. J Am Coll Cardiol.

[21] Ahn JM, Park DW, Kim YH (2012). Comparison of resolute zotarolimus-eluting stents and sirolimus-eluting stents in patients with de novo long coronary artery lesions: a randomized LONG-DES IV trial. Circ Cardiovasc Interv.

[22] Park KH, Ahn Y, Koh YY (2019). Effectiveness and safety of zotarolimus-eluting stent (Resolute™ Integrity) in patients with diffuse long coronary artery disease. Korean Circ J.

[23] Neupane S, Khawaja O, Edla S (2019). Meta-analysis of drug-eluting stents compared with bare metal stents in high bleeding risk patients undergoing percutaneous coronary interventions. Catheter Cardiovasc Interv.

[24] Lim S, Koh YS, Kim PJ (2016). Incidence, implications, and predictors of stent thrombosis in acute myocardial infarction. Am J Cardiol.

[25] Witzke CF, Martin-Herrero F, Clarke SC, Pomerantzev E, Palacios IF (2004). The changing pattern of coronary perforation during percutaneous coronary intervention in the new device era. J Invasive Cardiol.

[26] Shimony A, Zahger D, Van Straten M, Shalev A, Gilutz H, Ilia R, Cafri C (2009). Incidence, risk factors, management and outcomes of coronary artery perforation during percutaneous coronary intervention. Am J Cardiol.

